# Progress to Control and Eradication of Peste des Petits Ruminants in the Southern African Development Community Region

**DOI:** 10.3389/fvets.2019.00343

**Published:** 2019-10-15

**Authors:** Andrea Britton, Alexandre Caron, Berhanu Bedane

**Affiliations:** ^1^Ultimate Efficacy Consulting, Melbourne, VIC, Australia; ^2^ASTRE, Uni Montpellier, CIRAD, INRA, Montpellier, France; ^3^Faculdade de Veterinaria, Universidade Eduardo Mondlane, Maputo, Mozambique; ^4^FAO SFS, Harare, Zimbabwe

**Keywords:** peste des petits ruminants, Southern African Development Community, surveillance, risk-based approaches, small ruminants

## Abstract

In southern Africa, small ruminants are an important source of nutrition and income to resource-poor small holder farmers. After spreading from West to Central and Eastern Africa, peste des petits ruminants (PPR) emerged in the United Republic of Tanzania in 2008 and has since been reported in Angola, the Democratic Republic of the Congo, and the Comoros. The disease can cause considerable morbidity and mortality in naïve sheep and goat populations and severely impact rural livelihoods, particularly those of women. Gaps in the knowledge of PPR epidemiology still exist, particularly around the role of small-ruminant movement and the role of the abundant wildlife in southern Africa. The capacity of veterinary services to undertake surveillance and control PPR is heterogeneous within the region, with vaccination being limited. The Pan African strategy for the control and eradication of PPR mirrors the Global Strategy and provides the framework for the Southern African Development Community (SADC) region to meet the 2030 goal of eradication. Five countries and one zone within Namibia are officially PPR free according to OIE Standards. Most countries have developed national strategies for the control and eradication of PPR. To strengthen national and regional PPR eradication programme goals, there is a need for a regional risk-based surveillance adapted to infected, high-risk and lower-risk countries that will enable targeted and efficient control, rapid response to incursions and prevention of spread as well as improved preparedness. Continued international and national support will be necessary including laboratory diagnostics and enhancing surveillance capacity to prevent further spread southwards on the continent.

## Introduction

Peste des petits ruminants (PPR) is a World Organization for Animal Health (OIE) listed disease (1) caused by a morbillivirus resulting in variable respiratory and enteritis associated clinical disease in sheep and goat populations. PPR can also infect cattle, camels, domestic buffaloes, and wild ruminants (2). Given the high morbidity and mortality of PPR infection in immune-naïve small ruminants, the economic and food security impact of outbreaks is large for small-holder farmers. Women's livelihoods and resilience are particularly affected by PPR as women predominately rear small ruminants primarily for income generation and food security (3). The annual cost of PPR-associated sheep and goat deaths for worldwide infected countries is estimated between 794 million and 2.7 billion US dollars (4). This contagious viral disease has steadily expanded its geographical distribution from West into Eastern Africa and more recently to the Southern African Development Community (SADC) countries. Given the porous nature of country borders and movement of animals in many African countries, the risk of spread is high for countries bordering PPR infected ones.

Following the successful eradication of rinderpest globally in 2011, the Food and Agriculture Organization of the United Nation (FAO) and the OIE developed the Global Strategy for the Control and Eradication (GSCE) of PPR (5) to enable this plague to be the next eradicated animal disease by 2030. The control and eventual eradication of PPR will contribute significantly to achieving the elimination of poverty [Sustainable Development Goals (SDG 1)] and the end of hunger and malnutrition (SDG2) as well as contributing to other SDGs (3, 5, 8, 11, 12 and 17) (6). The global strategy was endorsed by 45 African Countries and the African Union Inter-African Bureau for Animal Resources (AU-IBAR) voiced its support for the global programme (7). The SADC region had already developed its own PPR control strategy (8). The global conference on “*Partnering and Investing for a Peste des Petits Ruminants Free World”* organized by the OIE and FAO in 2018, hosted by the European Commission was to reaffirm the political will of countries and to mobilize resources (6) to meet the 2030 eradication goal.

With the southern spread of this disease into the SADC region and issues associated with differentiating PPR from other diseases (9), national and regional approaches are urgently needed. SADC is the only region in sub-Saharan Africa with non-infected countries and therefore plays an important role in facilitating the control and eradication of PPR in infected countries which will in turn reduce the risk of disease spread further south on the African continent. An overview of the current situation is presented in this paper and the main constraints and opportunities to control PPR in the SADC regions are discussed.

## Situation Analysis

### Current PPR Status in SADC

**Figure 1:**
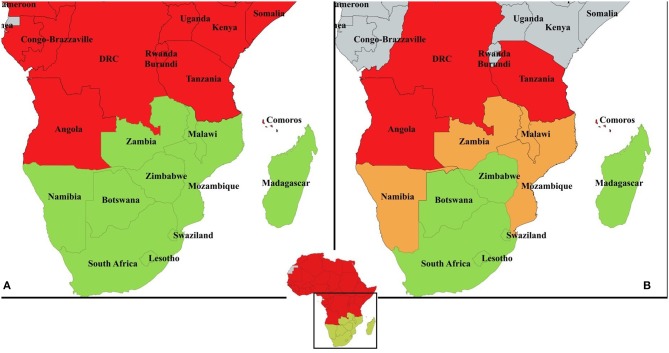
**(A)** PPR country data compiled from official reports and literature between 2008 and 2018. In red, countries with at least one occurrence of disease reported, in green countries with absence of disease or disease never been reported. Angola is considered infected in at least one zone by multiple references (see text) despite no OIE report of clinical disease. Zambia has reported seropositivity in 2015 to OIE but subsequent surveillance failed to prove occurrence of disease; **(B)** suggested risk-based approach for PPR surveillance and control in the Southern African Development Community: in red, “infected countries" with presence of the disease in at least one zone; in orange, “high-risk countries” sharing a border with an infected country; in green, “lower-risk countries” with no border shared with any infected country. Madagascar and Seychelles could be considered at risk because of the maritime trade of small ruminants with Comoros. However, the scale of this trade in intensity and frequency is not quantified. Mauritius is considered lower-risk country given its assumed low level of maritime trade with Comoros.

In southern Africa, PPR has spread into new areas in recent years (Figure 1A). Tanzania was first infected probably from imported animals from Kenya in 2008 and represents an important potential source of PPR viruses for the rest of the region (10). PPR is now considered endemic in Tanzania in small ruminants with PPR lineages II, III, and IV circulating (11). The disease has spread from Tanzania to the Democratic Republic of Congo (DRC) and Comoros (12, 13). Around 2012, Angola was infected probably with imported animals from DRC but these outbreaks have not been officially recorded (14). So far, no clinical disease has been reported in Namibia, Malawi, Mozambique, or Zambia (4). Zambia did detect PPR sero-positive goats in recent years, though in the absence of clinical disease, suggesting either that antibodies were from imported vaccinated animals or previously infected (i.e., from Tanzania and/or DRC) or false positives (Bedane personal communication, roadmap meeting). The situation in Mozambique is similar (15). The borders between Tanzania, DRC, and Angola and neighboring non-infected countries represent important entry gates for PPR into the rest of southern Africa. Namibia (Northern Communal Area), Malawi, Mozambique, and Zambia are therefore considered at high-risk of PPR infection. Botswana, Eswatini, Mauritius and South Africa and the southern zone of Namibia, are declared by the OIE as PPR free. Lesotho and Zimbabwe are also considered at lower-risk of PPR infection (Figure 1B). Madagascar and Seychelles could be considered at risk because of the maritime trade of small ruminants with Comoros (12). However, Madagascar has been declared PPR free by the OIE in 2018, where now efforts for surveillance need to be strengthened to avoid reversal of this status.

### Epidemiology of PPR in Southern Africa

PPR appears a good candidate for eradication according to criteria for eradication (16). However, the large populations of sheep and goats and their high population turnover (annual turnover rates of up to 30%) necessitate a higher effort (and costs) for control (14). The epidemiology of PPR is relatively well-defined but gaps in knowledge still exist and variability between regions may occur.

Firstly, following initial exposure of small-ruminant populations to PPR, a high mortality and morbidity is expected, which provides a visible clinical picture detectable by passive surveillance systems. However, in African small-scale farming systems, co-infection by multiple pathogens is frequent and could blur the expected clinical picture (17). PPR is also known to be a seasonal disease in some African endemic regions with peak infections usually occurring during the cool, dry season (18). This season in southern Africa starts in April and extends to August in some areas, providing a long environmental window for PPRV transmission. Under these climatic conditions, little is known about the PPRV persistence in urine and faeces (14).

Secondly, as in other African regions, the SADC region hosts countries with large small-ruminant populations (e.g., South Africa) and many are still free of PPR unlike all other African countries. Long-range cross-border trade of small ruminants involving two or more countries is frequent in the region (19). The patterns of this trade are largely unknown despite their relevance for the introduction and spread of animal diseases. Short-range trade (involving adjacent districts in neighboring countries), considered also to be illegal, represents an important socio-cultural component of local livelihoods. In addition, climate change is expected to increase extreme climatic events including droughts (20). Those events will affect mainly the poorest populations depending mostly on small ruminant production and living in the most arid areas. Droughts and political instability have been shown already to play their role in PPR spread (21).

Finally, some questions still remain on the host range of PPRV and their role in the local PPR epidemiology (22). In particular, little is known regarding virus excretion in infected camels, cattle, and wildlife (23, 24). In West Africa, cattle seem to be a dead-end host for PPR (25) but the role of local southern African breeds could be different (e.g., these breeds experienced different selective pressures by the rinderpest virus). In Africa, the role of most wild ungulate species in PPR epidemiology is largely unknown, as no clinical disease has ever been reported despite exposure (26). Clinical disease has been observed in African ungulates in zoo environments elsewhere (27) and in other wild ungulate species in central Asia (28, 29). The southern African region has large and healthy wildlife populations with relative freedom of movements across borders thanks to the creation of Transfrontier Conservation Areas (TFCAs) (30, 31). In addition, several species are endemic to the region (e.g., springbok) and some countries such as South Africa and Namibia have developed an important wildlife industry where animals maybe bred in conditions in-between natural and zoo settings where they could become particularly susceptible to PPR.

### Regional Capacity for PPR Surveillance and Control

The SADC Secretariat has identified PPR as one of the three major Transboundary Animal Diseases affecting regional and international trade (32). The SADC strategy (8) describes the limited PPR control capacity in SADC region in relation to diagnosis and surveillance, knowledge of virus transmission and susceptible species and differentiation of infected and vaccinated animals. Legislation on the use of PPR vaccines was also noted as an issue in most countries. The Pan African Veterinary Vaccine Center (AU-PANVAC) is mandated to provide quality assurance of all veterinary vaccines produced or imported into Africa and to coordinate the harmonization of veterinary vaccine registration with the support of the Global Alliance for Livestock Veterinary Medicines (GALVmed) and the OIE, which will be important for many SADC countries should they require PPR vaccine quickly due to an incursion.

Effective vaccination campaigns to ensure sustained herd immunity with 80 percent population coverage, will be pivotal to eradicating PPR as it was for eradicating rinderpest (14) though the high reproductive rate of small ruminants may warrant the need for annual vaccination in some flocks. Vaccination of small ruminants is limited in some areas due to the cost of vaccines, delivery and access to animals. Current vaccines against PPR virus are homologous vaccines (33) and require only one dose for life-long protection. The first vaccine was against lineage II (Nigeria 75/1) Africa PPR virus strain and has been used for 30 years. The impossibility to differentiate between vaccinated and infected animals and its thermolability are some of the limitations of this vaccine. Recent research on freeze-drying these types of live-attenuated vaccines have enabled thermostability and resistance to high temperatures in the field (34). The Botswana Veterinary Institutes (BVI) capacity in establishing and maintaining PPR-VAC^®^ was confirmed during a recent FAO supported project. This live-attenuated vaccine has also been assessed recently using an *in-vivo* challenge model in goats (35). Additionally, a thermo-adapted live-attenuated PPR vaccine has been trialed in goats in India (36). Assessment of the cross-lineage efficacy of different PPR vaccines is important given SADC has several lineages circulating (37). Recently comparative studies have indicated that the Nigeria 75/1 strain vaccines produce stronger antibody responses than the India S96, though the Indian strain vaccine elicits a greater cell-mediated immune response (38).

Given concurrent infection of sheep and goats with PPR and other diseases such as FMD or goat-pox (17, 39) a bivalent vaccine or concurrent vaccination would be of benefit to livestock owners and would be in line with the GSCE targeting other small ruminant diseases during the eradication programme. Development of vaccines based on Differentiating Infected from Vaccinated Animals (DIVA) technology will assist in surveillance and eradication (37). Unfortunately, these recombinant vaccines require booster doses and cost more than conventional vaccines but they have the advantage of temperature stability and their DIVA properties.

The SADC member states have a network of laboratories (provincial and national laboratories) for the surveillance of PPR and PPR diagnostic capacity which varies between countries. As in most national laboratories, the laboratories in Malawi, Mozambique, Namibia, Zambia, and Zimbabwe have been using c-ELISA assays to conduct PPR sero-surveillance in high-risk areas to detect the presence or absence of PPRV (Country reports, 2019). To increase the test sensitivity and affordability, AU-PANVAC has developed a blocking (b)-ELISA test (40). Some SADC countries have participated in the validation trial of this test (e.g., Malawi), others have been supplied kits (e.g., Mozambique) and others have requested them (Bedane personal communication). Most of the national laboratories have molecular PPR diagnostic capacity (e.g., PCR or qPCR). The capacity to conduct virus neutralization tests—OIE gold standard—and virus isolation and sequencing is absent from most national laboratories in SADC. Consequently, PPR confirmatory diagnostics of doubtful results requires countries to send samples to OIE reference laboratories for PPR (e.g., CIRAD or Pirbright Institute) (41) or to AU-PANVAC.

New field surveillance strategies may assist in the early diagnosis of disease and provide increased sensitivity and specificity of tests by targeting PPR virus specific antibodies, antigens, or genetic material (41–43). The direct detection of PPR virus genetic material and antigen in fecal samples could be used in small ruminant and wildlife surveillance (41). A pen-side test using quantum dots with a lateral-flow test strip has been evaluated in the field with similar results to c-ELISA (43). Such, a pen-side test that could confirm several small ruminant pneumo-enteritis diseases would be useful (34).

### FAO and OIE Guidance and Support

**Figure 2:**
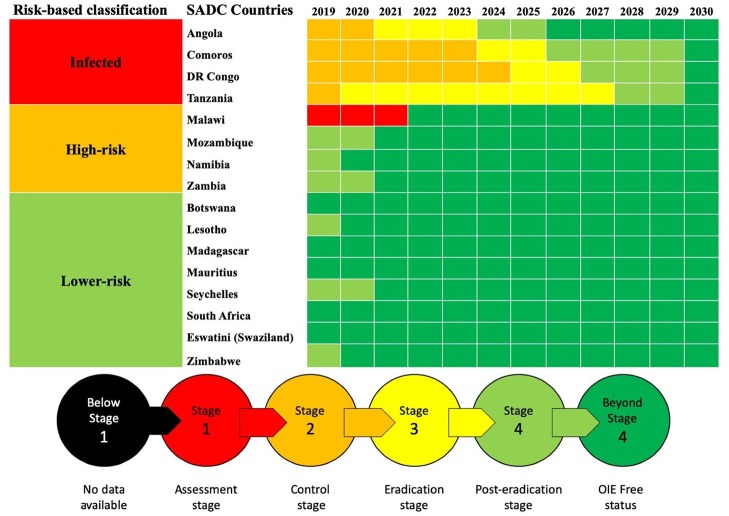
Progressive control pathway for SADC countries as reported during and following the Second Road Map Meeting, March 2019, Lusaka, Zambia.

FAO and OIE have established a Global Secretariat, which coordinates efforts for the PPR Global Eradication Programme (GEP) (44) based on a Progressive Control Pathway (PCP). The Global Secretariat is conducting Regional Roadmap workshops for the PCP implementation. In southern Africa, two Regional Roadmap meetings took place in October 2016 in Harare, Zimbabwe and in March 2019 in Lusaka, Zambia (45). The PPR Roadmap meetings ensure continuous evaluation and monitoring of the PPR situation and help in harmonizing policies and strategies among countries, as well as with other regions, for the implementation of the PPR GSCE. This strategy follows three core components advocating a risk-based approach to disease control to better target “virus hotspots.” The progressive stepwise approach—no available data (stage 1) to OIE free status (stage 5)—(Figure 2) and the PPR Monitoring and Assessment Tool (PMAT) are used in these meetings and correspond to a combination of decreasing levels of epidemiological risk and increasing levels of prevention and control capabilities.

The support provided by OIE and FAO both directly and indirectly assists SADC countries to progress along their respective PPR roadmap pathways (Figure 2). The FAO has been actively building capacity to prevent PPR introduction into Malawi, Mozambique, and Zambia through a Technical Cooperation Project 2013–2015 involving serological surveillance, local stakeholders awareness, and building rapid diagnostic capacity and national contingency and preparedness plans (TCP/SFS/3403) (46). Additional support by FAO provided to Madagascar and Lesotho enabled the former to obtain “Freedom from PPR certification” in 2018 while Lesotho will soon submit the documentation for its OIE freedom following FAO project TCP/LES/3604 (Pers. Com. Bedane). PPR control and eradication became one of the components of a recently launched SADC-based project financed by EU (“Support Toward the Operationalization of the SADC Regional Agricultural Policy”—GCP /SFS/004/EC). Additionally, OIE Performance of Veterinary Services (PVS) tool (47) will greatly support the assessment of the 47 Critical Competencies of Veterinary Services in countries and of areas specific to PPR control and eradication (7). Better control and diagnosis of other small ruminant diseases is also necessary for improving farmer participation. OIE has also been assisting to build PPR diagnostic capacity through focal point training, including fifth cycle workshop “Wildlife Health Information Management” 2018 and laboratory twinning projects between reference laboratories and SADC laboratories (e.g., in Tanzania).

## The Way Forward

Progress toward the control and eradication of PPR in SADC is now well-planned by many southern African countries. However, there is a need to coordinate efforts at the regional level. Three risk-based categories can be identified (infected, high-risk, lower-risk countries, Figure 1B). Better coordination between countries within the same category and between categories should improve harmonized surveillance and targeted control.

Following the PCP, a better understanding of the epidemiology of PPR in the region and its contributing factors will be necessary for eradication and this will require funding for field epidemiology research. Urgent active surveillance is required to establish the extent of PPR sero-positive areas in infected (across country) and high-risk (border areas) countries. In parallel, sheep and goat movements need to be better understood across the region. Studies on legal animal movement (e.g., by trucks or other vehicles) and other more informal cross-border movements will require participatory methods to enable mapping. A better understanding of cultural and social practices around small ruminant production systems of small-holder farmers in southern Africa is necessary in order to optimize surveillance and control of PPR (and other diseases). Women are known to be managing small ruminant production systems in Africa and, through communication and training tools, they should be empowered with the primary level of passive surveillance systems and control tools as identified in a study on gendered barriers to livestock vaccine uptake and ongoing gender inclusive vaccine study in Kenya (48, 49). Clarifying the role of wildlife and wildlife/livestock interfaces is also of paramount importance for SADC.

Risk-based approaches should be used to better understand the risks of introduction from infected to high-risk countries; the risk of disease spread once introduced into a new country and from there to other lower-risk countries. Spatial epidemiology can include different types of data layers such as the presence of wildlife populations, roads, density of small ruminants, each weighted by expert knowledge (50). These risk assessments are important to inform policy development, contingency planning, and for allocating scarce resources to high-risk areas within countries (13).

Veterinary services' capacity building is necessary in order to survey, control and eradicate PPR from SADC. In infected countries, going through stage 2–4 should be done through good communication with neighboring non-infected countries in order for them to survey for PPR with the most updated information. In high-risk countries, controlling animal movements is difficult with porous borders. Therefore, strategic passive surveillance for early detection (e.g., clinical and laboratory surveillance in markets or cross-border trade hubs) and early-response (e.g., vaccination) is needed to prevent outbreaks in new areas. The specific epidemiological context of SADC countries implies that surveillance systems should be prepared to expect non-conventional disease expression as the incursion of PPR in the Maghreb region showed moderate clinical signs and low rates of mortality. Improving biosecurity and sanitary protection through Public Private Partnerships will also be necessary (51) and FAO and OIE can help by facilitating donor agency-country relationships. Capacity building and experience sharing between infected and non-infected countries are important as demonstrated in FAO/OIE workshops. Countries at lower-risk of PPR introduction should get prepared using risk-based approaches at reacting to PPR outbreaks on their territory given their specific context (in particular given the size of the wildlife industry in some countries). Vaccines that are thermotolerant, produced in large quantities and if possible have DIVA abilities are needed. Further, PPR molecular diagnostic training and laboratory equipment and reagents are also needed in the region.

## Conclusion

The support of international organizations (i.e., FAO and OIE) and SADC technical committees will be of paramount importance to ensure effective regional collaboration. The experience from meetings and trainings organized by these groups has shown that trust and sustainable relationships between stakeholders and veterinary services is crucial to facilitate information flow within the region. The updated SADC strategy for the control and eradication of PPR will further guide regional coordination and provide leadership to meet the 2030 goal.

## Data Availability Statement

All datasets generated for this study are included in the manuscript/supplementary files.

## Author Contributions

AB and AC contributed equally to the design and writing of the manuscript. BB contribution to FAO activities, situation analysis of PPR in the region, laboratory capacity, and overall review of the manuscript.

### Conflict of Interest

The authors declare that the research was conducted in the absence of any commercial or financial relationships that could be construed as a potential conflict of interest.
